# Asthma Mortality Among Persons Aged 15–64 Years, by Industry and Occupation — United States, 1999–2016

**DOI:** 10.15585/mmwr.mm6702a2

**Published:** 2018-01-19

**Authors:** Opal Patel, Girija Syamlal, John Wood, Katelynn E. Dodd, Jacek M. Mazurek

**Affiliations:** ^1^Association of Schools and Programs of Public Health/CDC Public Health Fellowship Program; ^2^Respiratory Health Division, National Institute for Occupational Safety and Health, CDC.

In 2015, an estimated 18.4 million U.S. adults had current asthma, and 3,396 adult asthma deaths were reported (*1*). An estimated 11%–21% of asthma deaths might be attributable to occupational exposures (*2*). To describe asthma mortality among persons aged 15–64 years,* CDC analyzed multiple cause-of-death data^†^ for 1999–2016 and industry and occupation information collected from 26 states^§^ for the years 1999, 2003, 2004, and 2007–2012. Proportionate mortality ratios (PMRs)^¶^ for asthma among persons aged 15–64 years were calculated. During 1999–2016, a total of 14,296 (42.9%) asthma deaths occurred among males and 19,011 (57.1%) occurred among females. Based on an estimate that 11%–21% of asthma deaths might be related to occupational exposures, during this 18-year period, 1,573–3,002 asthma deaths in males and 2,091–3,992 deaths in females might have resulted from occupational exposures. Some of these deaths might have been averted by instituting measures to prevent potential workplace exposures. The annual age-adjusted asthma death rate** per 1 million persons aged 15–64 years declined from 13.59 in 1999 to 9.34 in 2016 (p<0.001) among females, and from 9.14 (1999) to 7.78 (2016) (p<0.05) among males. The highest significantly elevated asthma PMRs for males were for those in the food, beverage, and tobacco products manufacturing industry (1.82) and for females were for those in the social assistance industry (1.35) and those in community and social services occupations (1.46). Elevated asthma mortality among workers in certain industries and occupations underscores the importance of optimal asthma management and identification and prevention of potential workplace exposures.

National Vital Statistics System’s multiple cause-of-death data for 1999–2016 were analyzed to examine asthma mortality among persons aged 15–64 years. Asthma deaths were identified from death certificates using *International Classification of Diseases, 10th Revision* underlying cause-of-death codes J45 (asthma) and J46 (status asthmaticus). Death rates per 1 million persons aged 15–64 years by sex, race, ethnicity, and year were age-adjusted using the 2000 U.S. Census standard population. Time trends were assessed using a first-order autoregressive linear regression model to account for the serial correlation. Industry and occupation information available from 26 states for the years 1999, 2003, 2004, and 2007–2012^††^ was coded^§§^ using the U.S. Census 2000 Industry and Occupation Classification System. PMRs, adjusted by 5-year age groups and race, were generated by industry and occupation for males and females. In addition, 95% confidence intervals (CIs) were calculated assuming Poisson distribution of the data. Retired, unemployed, and nonpaid workers and those with information that was unknown or not reported for industry or occupation were excluded from PMR analyses.

During 1999–2016, a total of 33,307 U.S. decedents aged 15–64 years had asthma or status asthmaticus assigned as the underlying cause of death (Table 1) for an overall death rate of 8.89 per 1 million persons. The highest asthma death rates were among adults aged 55–64 years (16.32 per 1 million persons), females (9.95 per 1 million persons), persons who were not Hispanic or Latino (9.39 per 1 million), and blacks or African Americans (25.60 per 1 million persons). The age-adjusted asthma death rate per 1 million persons aged 15–64 years decreased 24.6% from 11.41 in 1999 to 8.60 in 2016 (p<0.01). The age-adjusted asthma death rates among females aged 15–64 years decreased from 13.59 per 1 million in 1999 to 9.34 in 2016 (p<0.001), and among males decreased from 9.14 (1999) to 7.78 (2016) (p<0.05). By state, annualized age-adjusted asthma death rates ranged from 4.59 per 1 million in Maine to 14.72 in the District of Columbia for males and from 6.70 per 1 million in North Dakota to 15.30 in Mississippi for females (Figure).

**TABLE 1:** Number of asthma deaths* and age-adjusted asthma death rates^†^ among persons aged 15–64 years, by sex and selected characteristics — United States, 1999–2016^§^

Characteristic	Males		Females		Overall	
	No. of deaths (% of asthma deaths)	Death rate	No. of deaths (% of asthma deaths)	Death rate	No. of deaths (% of asthma deaths)	Death rate
**Overall (% of all asthma deaths)**	14,296 (42.9)	7.78	19,011 (57.1)	9.95	33,307 (100.0)	8.89
**Age group (yrs)** ^¶^						
15–24	1,731 (12.1)	4.42	1,035 (5.4)	2.78	2,766 (8.3)	3.62
25–34	2,272 (15.9)	6.12	1,818 (9.6)	4.97	4,090 (12.3)	5.55
35–44	2,874 (20.1)	7.55	3,692 (19.4)	9.60	6,566 (19.7)	8.58
45–54	3,853 (27.0)	10.28	6,284 (33.1)	16.22	10,137 (30.4)	13.30
55–64	3,566 (24.9)	12.39	6,182 (32.5)	19.98	9,748 (29.3)	16.32
Race**						
American Indian or Alaska Native	138 (1.0)	6.28	198 (1.0)	9.15	336 (1.0)	7.75
Asian or Pacific Islander	525 (3.7)	5.67	439 (2.3)	4.23	964 (2.9)	4.92
Black or African American	5,695 (39.8)	25.21	6,463 (34.0)	25.76	12,158 (36.5)	25.60
White	7,938 (55.5)	5.28	11,911 (62.7)	7.74	19,849 (59.6)	6.52
**Ethnicity^††^**						
Hispanic or Latino	1,348 (9.4)	5.49	1,474 (7.8)	6.37	2,822 (8.5)	5.96
Not Hispanic or Latino	12,862 (90.0)	8.21	17,468 (91.9)	10.48	30,330 (91.1%)	9.39
Unknown	86 (0.6)	N/A	69 (0.4)	N/A	155 (0.5)	N/A
**Year**						
1999	824	9.14	1,257	13.59	2,081	11.41
2000	878	9.60	1,150	12.24	2,028	10.95
2001	792	8.47	1,192	12.41	1,984	10.49
2002	872	9.14	1,148	11.71	2,020	10.49
2003	828	8.54	1,162	11.62	1,990	10.12
2004	770	7.82	1,044	10.21	1,814	9.06
2005	720	7.21	1,102	10.59	1,822	8.96
2006	721	7.12	1,039	9.81	1,760	8.52
2007	745	7.22	908	8.51	1,653	7.89
2008	667	6.47	931	8.54	1,598	7.52
2009	699	6.69	996	9.08	1,695	7.92
2010	747	7.04	982	8.86	1,729	7.97
2011	732	6.82	953	8.45	1,685	7.67
2012	850	7.91	988	8.71	1,838	8.31
2013	852	8.01	999	8.77	1,851	8.43
2014	875	8.19	1,089	9.63	1,964	8.94
2015	885	8.14	997	8.65	1,882	8.43
2016	839	7.78	1,074	9.34	1,913	8.60
**p-value^§§^**	**0.72**	**<0.05**	**0.004**	**<0.001**	**0.11**	**<0.001**

**Abbreviation:** N/A = not available.

* Decedents who had *International Classification of Diseases, 10th Revision* codes J45 (asthma) or J46 (status asthmaticus) assigned as the underlying cause of death (i.e., the disease or injury that initiated the chain of morbid events leading directly to death, or the circumstances of the accident or violence that produced the fatal injury).

^†^ Age-adjusted asthma death rates per 1 million persons calculated using the 2000 U.S. Census standard population.

^§^ National Vital Statistics System. https://wonder.cdc.gov/.

^¶^ Age-specific asthma death rates per 1 million persons.

** Race and Hispanic origin are reported separately on the death certificate in accordance with standards set forth by the Office of Management and Budget. The American Indian or Alaska Native race category includes North, Central, and South American Indians, Eskimos, and Aleuts. The Asian or Pacific Islander race category includes Chinese, Filipino, Hawaiian, Japanese, and Other Asian or Pacific Islanders. https://wonder.cdc.gov/wonder/help/mcd.html.

^†† ^Deaths with Hispanic origin not stated are excluded from death rates calculation by Hispanic origin.

^§§^ For 1999–2016 linear time trend (examined using a first-order autoregressive linear regression model to account for the serial correlation).

**FIGURE:**
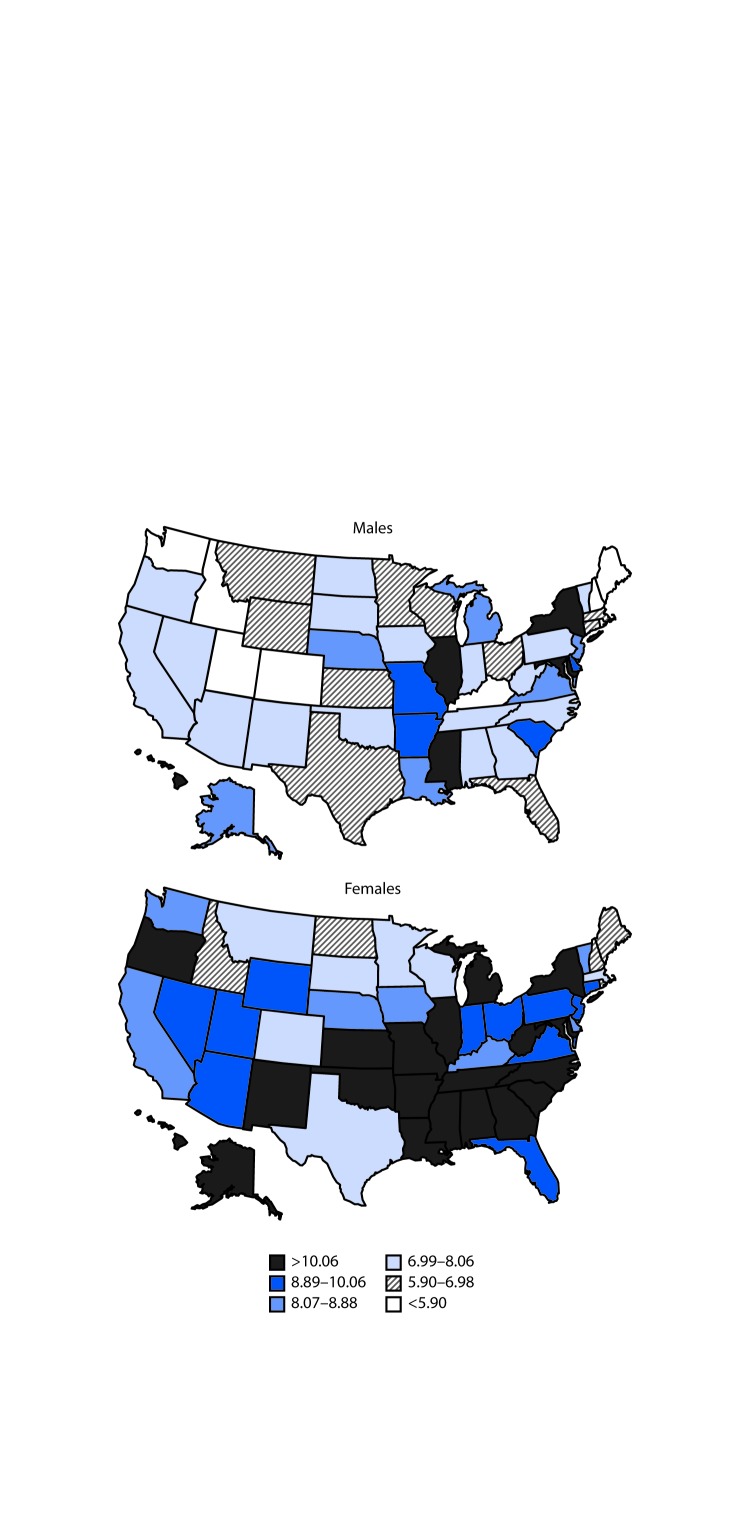
Annualized age-adjusted asthma death rate* per 1 million population aged 15–64 years,^†^ by sex and state^§^ — United States, 1999–2016^¶^ * Age-adjusted death rates were calculated by applying age-specific death rates to the 2000 U.S. Census standard population age distribution. https://wonder.cdc.gov/wonder/help/mcd.html#Age-AdjustedRates. ^†^ Decedents aged 15–64 years for whom the *International Classification of Diseases, 10th Revision* codes J45 (asthma) or J46 (status asthmaticus) were listed on death certificates as the underlying cause of death. ^§^ States represent the place of legal residence at the time of death. ^¶^ National Vital Statistics System. https://wonder.cdc.gov/.

Industry and occupation data were available for 3,393^¶¶^ (97.2%) of 3,491 asthma deaths, (1,398 of 1,435 [97.4%] males and 1,995 of 2,056 [97.0%] females) among persons aged 15–64 years that occurred in residents of 26 states during 1999, 2003, 2004, and 2007–2012 (Table 2). By industry, the highest number of asthma deaths occurred among males in the construction industry (184; 13.2% of asthma deaths in males) and among females in the health care industry (279; 14.0% of asthma deaths in females). By occupation, the highest number of asthma deaths occurred among male construction trades workers (149; 10.7%) and among female office and administrative support workers (186; 9.3%). By industry, PMRs were significantly elevated among males working in food, beverage, and tobacco products manufacturing (1.82; CI = 1.22–2.61), other retail trade (1.65; CI = 1.29–2.10), and miscellaneous manufacturing (1.45; CI = 1.13–1.86); and among females working in social assistance (e.g., individual and family services and child day care services) (1.35; CI = 1.00–1.79). By occupation, the PMR was significantly elevated among female community and social services workers (1.46; CI = 1.02–2.01).

**TABLE 2:** Industries and occupations with ≥25 asthma* deaths among persons aged 15–64 years, by sex — 26 states,^†^ 1999, 2003, 2004, and 2007–2012

Characteristic	No. of deaths	PMR^§,¶^ (95% CI)
**Industry**		
**Male (n = 1,079)**		
Food, beverage, and tobacco products manufacturing	29	1.82 (1.22–2.61)**
Other retail trade	69	1.65 (1.29–2.10)**
Miscellaneous manufacturing	66	1.45 (1.13–1.86)**
Arts, entertainment and recreation	29	1.30 (0.88–1.87)
Public administration	52	1.09 (0.83–1.45)
Health care	40	1.04 (0.74–1.42)
Repair and maintenance	46	1.01 (0.73–1.34)
Professional, scientific, technical and management services	34	1.00 (0.69–1.39)
Transportation and warehousing	89	0.98 (0.79–1.21)
Accommodation and food services	66	0.96 (0.75–1.23)
Educational services	29	0.95 (0.64–1.37)
Construction	184	0.92 (0.79–1.07)
Transportation equipment	28	0.78 (0.52–1.12)
Administrative and support, and waste management services	36	0.66 (0.46–0.91)
All other industries	282	—
**Female (n = 1,230)**		
Social assistance	49	1.35 (1.00–1.79)**
Arts, entertainment and recreation	26	1.29 (0.84–1.89)
Food and beverage stores	27	1.19 (0.78–1.73)
Private households	31	1.16 (0.79–1.64)
Health care	279	1.12 (1.00–1.27)
Other retail trade	96	1.10 (0.89–1.34)
Public administration	69	1.06 (0.83–1.35)
Accommodation and food services	116	1.01 (0.84–1.21)
Administrative and support, and waste management services	42	0.97 (0.70–1.31)
Transportation and warehousing	37	0.90 (0.63–1.24)
Finance and Insurance	48	0.90 (0.66–1.19)
Personal and laundry services	29	0.86 (0.58–1.24)
Educational services	94	0.85 (0.69–1.04)
Miscellaneous manufacturing	29	0.75 (0.50–1.07)
Professional, scientific, technical and management services	35	0.66 (0.46–0.92)
All other industries	223	—
**Occupation**		
**Male (n = 1,087)**		
Office and administrative support occupations	62	1.25 (0.97–1.61)
Other production occupations, including supervisors	51	1.21 (0.91–1.61)
Sales and related occupations	89	1.17 (0.95–1.45)
Laborers and material movers, hand	92	1.09 (0.88–1.34)
Motor vehicle operators	74	1.07 (0.85–1.36)
Metal workers and plastic workers	35	0.95 (0.66–1.33)
Food preparation and serving related occupations	46	0.91 (0.66–1.21)
Construction trades workers	149	0.89 (0.76–1.05)
Management occupations, except agricultural	61	0.89 (0.69–1.15)
Building and grounds cleaning and maintenance occupations	54	0.88 (0.67–1.16)
Electrical equipment mechanics and other installation, maintenance, and repair workers	26	0.85 (0.56–1.25)
Vehicle and mobile equipment mechanics, installers, and repairers	32	0.82 (0.56–1.15)
All other occupations	316	—
**Female (n = 1,239)**		
Community and social services occupations	36	1.46 (1.02–2.01)**
Laborers and material movers, hand	47	1.19 (0.88–1.59)
Healthcare support occupations	110	1.15 (0.95–1.39)
Food preparation and serving related occupations	100	1.12 (0.92–1.37)
Personal care and service occupations	75	1.09 (0.87–1.38)
Sales and related occupations	134	1.09 (0.92–1.30)
Health diagnosing and treating practitioners and technical occupations	59	1.00 (0.77–1.31)
Building and grounds cleaning and maintenance occupations	62	1.00 (0.78–1.30)
Management occupations, except agricultural	85	0.99 (0.80–1.24)
Business operations specialists	25	0.96 (0.62–1.42)
Education, training, and library occupations	70	0.93 (0.73–1.18)
Health technologists and technicians	28	0.91 (0.61–1.32)
Office and administrative support occupations	186	0.90 (0.77–1.04)
All other occupations	222	—

**Abbreviations:** CI = confidence interval; PMR = proportionate mortality ratio.

* Decedents who had the *International Classification of Diseases, 10th Revision* codes J45 (asthma) or J46 (status asthmaticus) assigned as the underlying cause of death (i.e., the disease or injury that initiated the chain of morbid events leading directly to death, or the circumstances of the accident or violence that produced the fatal injury).

^†^ Colorado, Florida, Georgia, Hawaii, Idaho, Indiana, Kansas, Kentucky, Louisiana, Michigan, Nebraska, Nevada, New Hampshire, New Jersey, New Mexico, North Carolina, North Dakota, Ohio, Rhode Island, South Carolina, Texas, Utah, Vermont, Washington, West Virginia, and Wisconsin. States represent the state where the death took place.

^§^ PMR is defined as the observed number of deaths from asthma in a specified industry/occupation, divided by the expected number of deaths from asthma. The expected number of deaths is the total number of deaths in industry or occupation of interest multiplied by a proportion defined as the number of asthma deaths in all industries or occupations, divided by the total number of deaths in all industries/occupations. The asthma PMRs were internally adjusted by 5-year age groups and race. CIs were calculated assuming Poisson distribution of the data.

^¶^ Retired, unemployed, and unpaid (229 males and 687 females) and unknown or not reported (90 males and 78 females) workers in industries, and retired, students, volunteers, homemakers and unemployed (233 males and 688 females) and unknown or not reported (78 males and 68 females) occupations were excluded from PMR analyses.

** Statistically significant elevated PMR.

## Discussion

The annual number of asthma deaths among persons aged 15–64 years has declined significantly from 1999 through 2016, most likely reflecting improvements in asthma management and effectiveness of prevention efforts (*3*,*4*). For example, replacing powdered latex gloves with powder-free natural rubber latex or nonlatex gloves reduced latex allergen exposure and substantially reduced work-related asthma*** among health care workers (*4*). Differences in asthma mortality by age, sex, and race/ethnicity have been previously reported (*5*). Based on an estimate that 11%–21% of asthma deaths might be attributable to occupational exposures (*2*), an estimated 3,664–6,994 asthma deaths during 1999–2016 (1,573–3,002 among males and 2,091–3,992 among females) might have been job-related, and therefore potentially preventable.

Female workers in the health care industry and male workers in the construction industry accounted for the highest industry-related numbers of asthma deaths. The PMRs were significantly elevated among males in the food, beverage, and tobacco products manufacturing, other retail trade, and miscellaneous manufacturing industries; and among females in the social assistance industry and in the community and social services occupations. A higher proportion of females with current asthma and a high frequency of exposures associated with work-related respiratory diseases have been observed in the health care and social assistance industries (*6*,*7*). National survey data indicate that approximately 9.1% (1.3 million) of 13.9 million female workers in the health care and social assistance industries, and 4.2% (394,000) of 9.4 million male workers in the construction industry, have current asthma.^†††^ Approximately 13.4% of health care and social assistance workers, 51.1% of construction workers, 31.8% of food manufacturing workers, 36.1% of beverage and tobacco product manufacturing workers, 40.0% of miscellaneous manufacturing workers, 21.5% of retail trade workers, and 3.7% of community and social services workers are frequently exposed to vapors, gas, dust, or fumes in the workplace (*6*). Workplace exposures to asthma-causing agents,^§§§^ such as cleaners, disinfectants, antibiotics, natural rubber latex among health care workers, and welding fumes and isocyanates (e.g., paints) among construction workers^¶¶¶^ have been associated with work-related asthma (*8*,*9*). Higher PMRs in certain groups might also be explained in part by workers leaving employment in industries and occupations with workplace exposures that exacerbate their asthma and moving to jobs with fewer workplace exposures (*10*). Likewise, retired, unemployed, and nonpaid workers might have left the workforce because of workplace exposures.

Differences in asthma mortality by industry and occupation underscore the need for identifying workplace exposures, early diagnosis, and treatment and management of asthma cases, especially among industries and occupations with higher mortality. Pharmaceutical treatment of asthma related to occupational exposures is similar to that for asthma that is not work-related (*3*). Early identification and elimination of exposures is the preferred means of primary prevention to reduce asthma related to occupational exposures; however, reduction of exposure might be considered when elimination of exposures is not possible (*4*). Establishing an accurate diagnosis and recommending appropriate management for workers with asthma related to occupational exposures is necessary to improve outcomes and could prevent asthma deaths (*4*).

The findings in this report are subject to at least five limitations. First, asthma and status asthmaticus diagnoses could not be validated. It is possible that some decedents were misdiagnosed. However, given the potential impact of asthma diagnosis and status asthmaticus on patients’ lives, it seems likely that asthma would be accurately recorded on death certificates. Second, no information was available to assess whether workplace exposures triggered asthma attacks that led directly to death. Some attacks might have been triggered by exposures outside of the work environment. Third, to the extent that asthma attacks were triggered by workplace exposures, industry and occupation information reported on death certificates might not be the industry and occupation in which workplace exposures actually occurred because guidelines for reporting industry and occupation on death certificates**** instruct recorders to report decedent’s “usual” industry and occupation (i.e., “the type of job the individual was engaged in for most of his or her working life”). Fourth, no work history was available to assess changes in employment. Retired and unemployed persons might have left the workforce because of severe asthma in relation to work. Finally, information on industry and occupation might not be nationally representative because only selected states provided information on industry and occupation, and only for certain years.

Effective asthma management tools are available from CDC at https://www.cdc.gov/asthma/tools_for_control.htm, and information on the evaluation and treatment of asthma is available from the American Thoracic Society at https://www.thoracic.org/statements/allergy-asthma.php. Additional guidance for diagnosing work-related asthma is available from the Occupational Safety and Health Administration at https://www.osha.gov/SLTC/occupationalasthma/. The elevated asthma mortality among workers in certain industries and occupations underscores the importance of optimal asthma management, and identification and elimination or reduction of potential workplace exposures (*3*,*4*,*9*).

**Summary**

**What is already known about this topic?**

In 2015, a total of 3,396 asthma deaths were reported among adults aged ≥18 years in the United States. An estimated 11%–21% of asthma deaths might be attributable to occupational exposures. Asthma deaths are preventable with proper asthma management and rapid response to asthma attacks.

**What is added by this report?**

Among U.S. adults aged 15–64 years, 33,307 deaths from asthma occurred during 1999–2016, including an estimated 3,664–6,994 (approximately 204–389 annually) that could be attributable to occupational exposures and were therefore potentially preventable. The highest asthma death rates were among adults aged 55–64 years, females, persons who were not Hispanic or Latino, and blacks or African Americans. By industry, asthma mortality was significantly elevated among males in food, beverage, and tobacco products manufacturing, other retail trade, and miscellaneous manufacturing, and among females in social assistance. By occupation, asthma mortality was significantly elevated among females in community and social services.

**What are the implications for public health practice?**

Elevated asthma mortality among male and female workers in certain industries and occupations highlights the importance of optimal asthma management, and identification and prevention of workplace exposures.

## Footnotes

* https://www.dol.gov/general/topic/youthlabor/agerequirements.

† Decedents who had the *International Classification of Diseases, 10th Revision* codes J45 (asthma) or J46 (status asthmaticus) assigned as the underlying cause of death (the disease or injury that initiated the chain of events that led directly and inevitably to death). https://wonder.cdc.gov/.

§ Colorado, Florida, Georgia, Hawaii, Idaho, Indiana, Kansas, Kentucky, Louisiana, Michigan, Nebraska, Nevada, New Hampshire, New Jersey, New Mexico, North Carolina, North Dakota, Ohio, Rhode Island, South Carolina, Texas, Utah, Vermont, Washington, West Virginia, and Wisconsin. States represent the state where the death took place.

¶ PMR was defined as the observed number of deaths from asthma in a specified industry/occupation, divided by the expected number of deaths from asthma. The expected number of deaths was the total number of deaths in industry or occupation of interest multiplied by a proportion defined as the number of asthma deaths in all industries and/or occupations, divided by the total number of deaths in all industries/occupations. The asthma PMRs for each sex were internally adjusted by 5-year age groups and race.

** Age-adjusted death rates were calculated by applying age-specific death rates to the 2000 U.S. Census standard population age distribution. https://wonder.cdc.gov/wonder/help/mcd.html#Age-AdjustedRates.

†† Information on industry and occupation was available only for decedents from 26 states for the years 1999, 2003, 2004, and 2007–2012. https://www.cdc.gov/niosh/topics/noms/default.html.

§§ https://webappa.cdc.gov/ords/norms-glossary.html#ind-occ.

¶¶ For 98 residents of these 26 states, deaths occurred in states that did not provide the industry and occupation information to the National Institute for Occupational Safety and Health. Retired, unemployed, and non-paid (229 males and 687 females) and unknown or not reported (90 males and 78 females) industries, and retired, students, volunteers, homemakers and unemployed (233 males and 688 females) and unknown or not reported (78 males and 68 females) occupations were excluded from PMR analyses.

*** Work-related asthma includes occupational asthma (i.e., new-onset asthma caused by factors related to work) and work-exacerbated asthma (i.e., preexisting or concurrent asthma worsened by factors related to work). https://www.cdc.gov/niosh/topics/asthma/default.html.

††† https://wwwn.cdc.gov/eworld/Grouping/Asthma/97.

§§§ Association of Occupational and Environmental Clinics list of occupational asthmagens. http://www.aoecdata.org/ExpCodeLookup.aspx.

¶¶¶ Occupational Safety and Health Administration. Health Hazards in Construction. https://www.osha.gov/dte/grant_materials/fy09/sh-19495-09/health_hazards_workbook.pdf.

**** https://www.cdc.gov/nchs/data/misc/hb_occup.pdf.
